# Protein trafficking across the cell envelope of gram-positive bacteria

**DOI:** 10.1128/jb.00100-25

**Published:** 2025-08-11

**Authors:** Dipanwita Bhattacharya, Ran Zhang, Wenqi Yu

**Affiliations:** 1 Department of Molecular Biosciences, College of Arts and Sciences, University of South Florida 7831, Tampa, Florida, USA; 2 Center for Antimicrobial Resistance, University of South Florida, Tampa, Florida, USA; University of Notre Dame, Notre Dame, Indiana, USA

**Keywords:** protein secretion, gram-positive bacteria, sec secretion, signal peptide, signal peptidase, signal peptide peptidase, extracellular chaperones and proteases, PrsA, HtrA, sortase, *Bacillus subtilis*, *Staphylococcus aureus*

## Abstract

Protein trafficking from the cytosol to specific sites of action is a fundamental process for bacterial survival and growth. In the monoderm gram-positive bacteria, this process involves traversing the cytoplasmic membrane, the membrane-wall interface, and the thick cell wall. With a primary focus on *Bacillus subtilis* and *Staphylococcus aureus*, this minireview aims to provide an overview and mechanistic insights into (i) protein translocation across the cytoplasmic membrane via the Sec system, (ii) protein processing by chaperones and proteases in the membrane-wall interface, and (iii) protein attachment to the cell wall and translocation across the cell wall. Furthermore, we emphasize recent advancements in spatial regulation of protein translocation systems. By examining conserved and unique mechanistic features, this review aims to offer a systematic understanding of protein trafficking in gram-positive bacteria.

## INTRODUCTION

Protein transport from within the cell to its site of action is a fundamental and essential cellular process. In parallel to the studies in eukaryotes, the inquiries of how bacteria export proteins began in the 1970s, with the demonstrations of cleavable signal sequences in exported proteins (1–4). Targeting the cytoplasmic protein β-galactosidase (encoded by *lacZ*) to membrane by fusing it with signal sequences of exported proteins not only supported the signal hypothesis of Blobel and Dobberstein (5, 6) but also provided powerful tools for elegant genetic screens that identified six *sec* genes (ABDEFY) in *Escherichia coli* (7–14). Combined with biochemical approaches of *in vitro* protein translocation systems (4, 15), the Sec secretion components from *E. coli* were identified and characterized between 1981 and 1993 (16). At the same time, the gram-positive bacteria, especially bacilli, were used to produce exoenzymes of industrial importance, but relatively much less was known about their protein secretion process (17, 18). It was thought that gram-positive bacteria might have a fundamentally different secretion apparatus. Earlier attempts to isolate proteins that interact with peptide-secreting ribosomes identified S (secretory) complex in *Bacillus subtilis* and membrane-bound ribosome protein complex in *Staphylococcus aureus* (19, 20), which, however, were later found to be pyruvate dehydrogenase (21). The *secY and secA* genes from *B. subtilis* were eventually identified by sequence homology analysis and DNA hybridization with the *E. coli* probes, revealing the conservation of Sec secretion machinery between gram-negative and gram-positive bacteria (22–25).

While the initial protein transport system across the cytoplasmic membrane is highly conserved across bacterial species, the steps of post-membrane translocation differ due to the difference in cell envelope structures in gram-negative and gram-positive bacteria. Gram-negative bacteria possess an inner cytoplasmic membrane and a lipopolysaccharide-rich outer membrane separated by a thin peptidoglycan layer (26). Gram-positive bacteria have only one cytoplasmic membrane (termed monoderm) surrounded by a thick cell wall peptidoglycan layer with immobilized teichoic acids and extracellular polysaccharides (27). The structure of cell wall peptidoglycan is conserved in bacteria, which is composed of repeating units of N-acetyl glucosamine (GlcNAc) and N-acetyl muramic acid (MurNAc) linked by β-1,4 glycosidic bonds. The linear glycan chains are cross-linked via stem peptides that are attached to MurNAc. The stem peptide cross-link varies among bacteria (28). Many gram-positive bacteria contain branched-stem peptides. *S. aureus*, for example, contains a pentaglycine branch linked to the third amino acid of the stem peptides. The branched-stem peptides serve as attachment sites for covalently anchored surface proteins (discussed in section “Covalent Binding to Cell Wall: the Act of Sortase”). Teichoic acids are major components of the gram-positive bacterial cell envelope, which includes wall teichoic acids (WTAs) and lipoteichoic acids (LTAs). WTAs are commonly composed of a disaccharide linkage unit and polyribitol phosphate or polyglycerol phosphate repeating units (29). WTAs are covalently attached to peptidoglycan and extrude the cell surface. LTAs are glycerol-phosphate polymers tethered to the cytoplasmic membrane via a glycolipid anchor, which is embedded underneath the peptidoglycan layer (30–32). Both WTAs and LTAs are modified by glycosylation and D-alanylation. While most gram-positive bacteria do not appear to have a clearly defined periplasmic space by conventional transmission electron microscopy, the development of cryo-electron microscopy of vitreous section technology revealed a bipartite cell envelope structure in *B. subtilis* and several other gram-positive bacteria (33–36). The structure consists of a low-density inner wall zone (IWZ, 22.3 nm wide in *B. subtilis*) and a high-density outer wall zone (33.3 nm wide in *B. subtilis*). Subsequent studies have suggested that LTA, but not peptidoglycan, is the major component of IWZ (37). A periplasmic-like space was proposed to exist in gram-positive bacteria, which was hidden by fixation and dehydration in the conventional electron microscopy method. While the function and structure of a periplasmic-like space in gram-positive bacteria remain to be further investigated, we will use the term “membrane-wall interface” for the space between the cytoplasmic membrane and the cell wall in this minireview.

Based on the cell envelope structure, protein trafficking in gram-positive bacteria involves translocation from the cytosol to the cytoplasmic membrane, the membrane-wall interface, the cell wall, and extracellular environment. In general, proteins that are targeted to distinct cellular compartments distinguish themselves by carrying specific targeting signals. There are mainly three pathways that transport proteins from the cytosol to the cytoplasmic membrane: the Sec, the signal recognition particle (SRP), and the twin-arginine translocation (TAT) systems. SecA-dependent Sec pathway is the major route for transporting proteins across the cytoplasmic membrane. The SRP pathway co-translationally transports mainly membrane proteins, and the TAT pathway transports folded proteins (38). Some gram-positive bacteria possess additional SecA and SecY paralogs, namely, SecA2 and SecY2, that serve crucial roles in translocating specific proteins and virulence factors (39). Lipoproteins are a group of proteins that carry a conserved lipobox and are attached to the outer leaflet of the membrane via the lipid moiety at their N-terminal cysteine (40). Upon membrane translocation, surface protein precursors carrying C-terminal cell wall sorting signals are recognized by the membrane-bound transpeptidase sortase and are covalently attached to the cell wall peptidoglycan (41). Some gram-positive bacteria also have specialized cargo translocation systems, including type III-like/flagellar systems, type IV/conjugation systems, and type VII secretion systems, which primarily transport pathogenic effector proteins and DNA (42–44).

**Fig 1:**
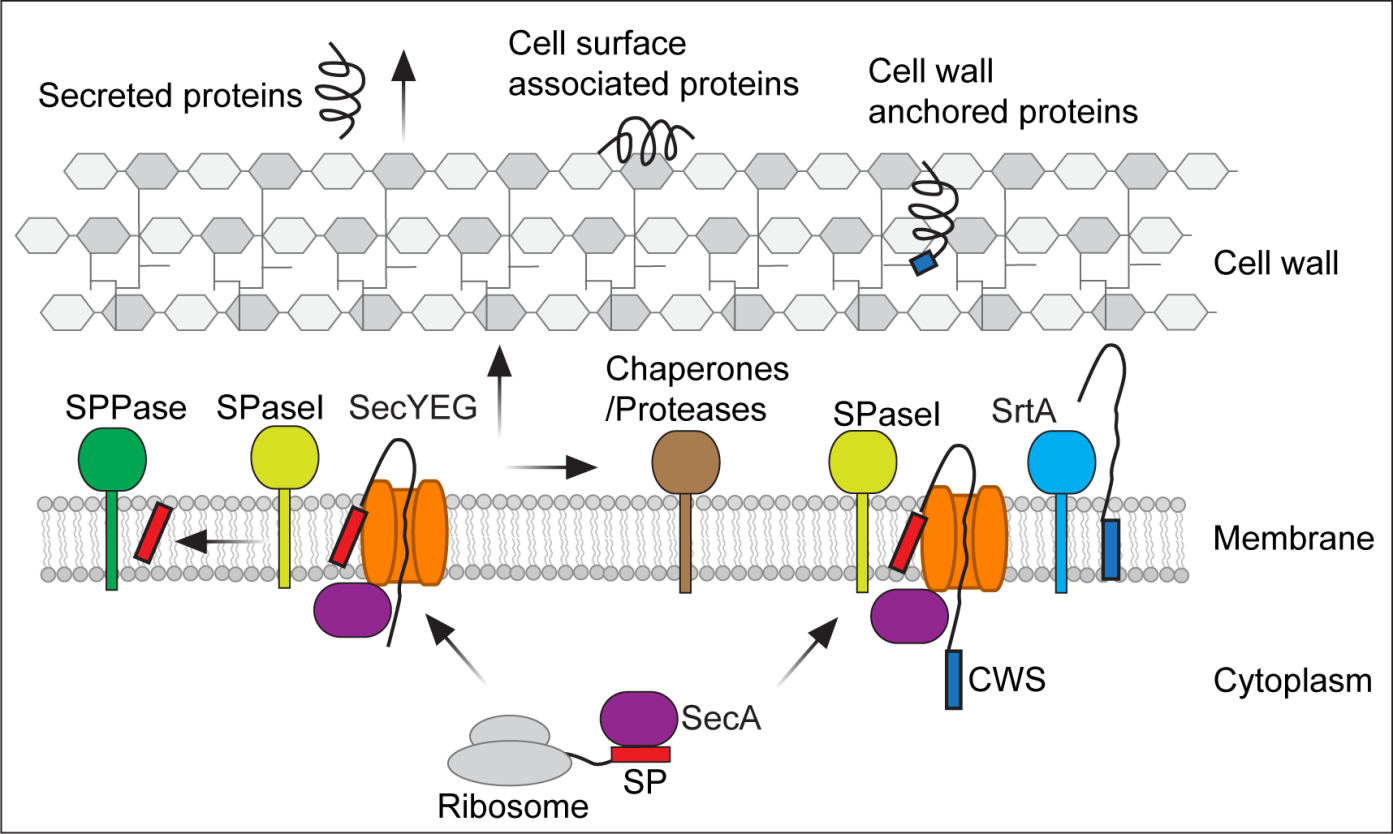
An overview of protein trafficking in gram-positive bacteria. The figure illustrates the protein secretion and anchoring process in gram-positive bacteria, demonstrating pathways including the Sec system, extracellular chaperones and proteases, and surface protein anchoring mediated by sortase. Abbreviations: CWS, cell wall sorting signal; SP, signal peptide; SPase I, type I signal peptidase; SPPase, signal peptide peptidase; SrtA, sortase A.

In this minireview, we aim to provide a systematic overview of the protein trafficking process across the cell envelope of gram-positive bacteria. We will focus on the SecA-dependent general Sec system that translocates the vast majority of secreted proteins but not specific protein translocation systems. Sequentially, we will explore (i) protein translocation across cytoplasmic membrane via the SecA-dependent Sec pathway, (ii) protein processing by chaperones and proteases in the membrane-wall interface, and (iii) protein trafficking through the cell wall or attachment to the cell surface. The trafficking pathways are illustrated in Fig. 1. The key components in these processes are summarized in Table 1. Furthermore, we summarize recent advances in spatial regulation of protein translocation systems and their roles in bacterial cell biology. We emphasize *Bacillus subtilis* and *Staphylococcus aureus*, with the former being a gram-positive model organism and industrial workhorse for secreted enzyme production and the latter being a representative of gram-positive pathogens. Homologous counterparts in gram-negative *E. coli* and other gram-positive bacteria are discussed, with which we highlight the conservation and diversity across bacterial species. “Diderm” gram-positive bacteria, such as *Corynebacterium* and *Mycobacterium*, will not be discussed in detail since they have complex cell envelope structures that reassemble both gram-positive and gram-negative bacteria.

**TABLE 1:** List of protein translocation components in gram-positive bacteria

Proteins		*E. coli* (gram- negative)	*B. subtilis* 168	*S. aureus* (NCTC 8325)	Homologs in other gram-positive bacteria	Function
Sec components	SecA	SecA	SecA	SecA	Conserved across species	ATPase and translocation motor, recognizes signal peptide on the nascent polypeptide, target and translocate pre-proteins to Sec translocon
	SecB	SecB	No SecB and CsaA (functional homolog of SecB)	No homolog	No homolog	Cytoplasmic chaperone, prevents proteins from aggregating and helps target them to the SecA/YEG translocon
	SecY	SecY	SecY	SecY	Conserved across species	Component of translocation channel, transports protein across the cytoplasmic membrane
	SecE	SecE	SecE	SecE	Conserved across species	Component of translocation channel, transports protein across the cytoplasmic membrane
	SecG	SecG	SecG	SecG	Conserved across species	Component of translocation channel, transports protein across the cytoplasmic membrane
	SecD	SecD	SecDF	SAOUHSC_01746 (homolog of SecDF)	Conserved across species	Protein translocase subunit, facilitates the transport of the translocating protein across the membrane
	SecF	SecF				Protein translocase subunit, facilitates the transport of the translocating protein across the membrane
	YajC	YajC	YrbF	SAOUHSC_01747 (YajC homolog)	*Streptococcus pneumoniae* (YajC) and *Lactococcus lactis* (YwaB)	Protein translocase accessory subunit, interacts with SecDF, facilitates proper insertion and translocation of the protein inside the membrane
Type I signal peptidase		LepB	SipS/T/U/V/W	SpsA (inactive) and SpsB	Conserved across species	Peptidase that cleaves the signal peptide from the pre-proteins
Signal peptide peptidase		RseP	RasP	SAOUHSC_01239 (RasP homolog)	*Listeria monocytogenes* (RseP), *Enterococcus faecalis* (RseP), and *Clostridium perfringens* (RasP)	Transmembrane protease that cleaves the remnants of signal peptides after their cleavage by the signal peptidase
		SppA	SppA	Not known	*Enterococcus faecium* (SppA)	
		Not known	TepA	Not known	Not known	
Membrane-wall interface processors	Chaperone	SurA (PrsA homolog)	PrsA	PrsA	*Bacillus anthracis* (PrsAA, PrsAB, and PrsAC); *L. monocytogenes* (PrsA1 and PrsA2); *S. pneumoniae* (PrsA); *Streptococcus pyogenes* (PrsA1 and PrsA2); *E. faecalis* (PrsA); and *L. lactis* (PmpA)	Extracellular chaperone helps folding of proteins post-membrane translocation
	Disulfide oxidoreductase	DsbA	BdbA/B/C	SAOUHSC_02694 (DsbA-related protein)	*L. monocytogenes* (DsbA family protein) and *E. faecalis* (DsbA-like protein)	Forms disulfide cross-link in proteins post-membrane translocation as part of folding before secretion
	HtrA family protease and chaperone	DegP (HtrA family protein)	HtrA and HtrB	SAOUHSC_01838 (HtrA1) and SAOUHSC_00958 (HtrA2)	*S. pneumoniae* (HtrA), *S. pyogenes* (HtrA), and *L. lactis* (HrtA)	Serine protease, degrades or refold misfolded or abnormally exported proteins and plays a crucial role in secretion stress response
Cell wall anchors	Sortase	Not known	YhcS and YwpE	SrtA and SrtB	*B. anthracis* (SrtA/B/C/D) and *E. faecalis* (SrtA/C)	Transpeptidase, anchors secreted proteins to the cell surface

## TRANSLOCATION ACROSS THE CYTOPLASMIC MEMBRANE VIA SECA-DEPENDENT SEC PATHWAY: FIRST GATEWAY AFTER SYNTHESIS

The recognition and targeting of the pre-proteins start at an early stage of synthesis, where several targeting factors accumulate near the exit of the ribosome and interact with the N-terminal domain of the newly generated pre-protein, as first proposed by Milstein et al. in 1972 (45). The target factors guide the pre-proteins to the membrane translocation apparatus where they are processed. In this section, we will describe the SecA-dependent Sec translocation system.

### Recognition of the pre-protein via signal peptides

The concept of signal sequences at the N-termini of pre-proteins was first introduced by Sabatini and Blobel in 1970 (46), who proposed that these sequences function as “zip codes” directing proteins to their various subcellular locations. These sequences are short stretches of amino acids located at the amino termini of pre-proteins (47). They can also bind to chaperones in the cytosol, preventing premature folding of pre-proteins before translocation (47–49). The receptors of the secretion system recognize the signal peptides and transfer the signal peptide-bearing pre-proteins to the translocation machinery. They serve as topological determinants for pre-proteins, initiating the translocation of the C-terminal hydrophilic region through the membrane, while the N-terminal part of the signal sequence remains attached to the cytoplasmic side of the membrane (50). The signal sequences in gram-positive bacteria are relatively longer than those in gram-negative bacteria and exhibit greater hydrophobicity (51). Despite these differences, they are structurally similar across species containing a short basic N region, a hydrophobic H region, and a C region that serves as the recognition site for the signal peptidase (SPase) (48). The enzyme SPase cleaves the signal sequence at specific sites characterized by amino acids with short side chains at the −3 and −1 positions relative to the scissile bond. The cleavage site is conserved in gram-positive and gram-negative organisms, represented by the alanine-X-alanine motif (47). Signal peptides with specific features target themselves for specific translocation systems. For example, the Tat signal peptides are characterized by a consensus twin arginine motif (S/T-R-R-X-F-L-K) at their N-termini, followed by a less hydrophobic H region (52, 53). Bacterial lipoprotein signal peptides share a similar tripartite “N-H-C” structure but are characterized by a shorter H region and a distinctive recognition C-terminal signal sequence, leucine-X-X-cysteine, which is recognized by type II SPase (54). Furthermore, many surface protein precursors contain a highly conserved “YSIRK/G-S” motif in their signal peptides (55), which we will discuss in detail in the section of “YSIRK/G-S Signal Peptide-Mediated Septal Trafficking”. Apart from these specific signal peptides, most Sec signal peptides are conserved with a tripartite N-H-C structure but vary significantly in their amino acid sequences and possess different capacities to secrete pre-proteins (56). Significant effort has been made to optimize signal peptides to increase protein secretion efficiency, and individual protein-based screening assays turned out to be the most successful (57).

### The general Sec secretory pathway

The general secretory system (Sec pathway) is the major route of protein transport across the cytoplasmic membrane (58). The core components of the Sec pathway are conserved across bacterial species, whereas diversified enzymes and paralogs with distinct structures and functions have been found in different gram-positive species (16).

#### Cytoplasmic chaperones and SecA, a ubiquitous component of the Sec translocons

Secretory pre-proteins with signal peptides are synthesized by the ribosome and directed to the Sec machinery. After synthesis, the pre-proteins interact with the cytoplasmic chaperones (CsaA, GroEL/ES, and DnaK-DnaJ-GrpE), either on the ribosome or in the cytoplasm (59, 60). Gram-positive and gram-negative bacteria differ notably in the cytoplasmic chaperone systems. In gram-negative bacteria, pre-proteins interact with either SecB or other chaperones such as trigger factor, DnaK, and GroEL (61, 62). Gram-positive bacteria generally lack a SecB homolog. However, *B. subtilis* utilizes CsaA as an alternative cytoplasmic chaperone for SecA-dependent pre-protein delivery (63, 64). It is important to note that CsaA and SecB do not share the same conserved binding region on SecA, indicating their different modes of action (64). CsaA is predominantly found in *B. subtilis* and a limited number of archaeal species. Suppressing CsaA is detrimental to the secretion of certain substrates, suggesting its role in transporting specific protein substrates (65). However, CsaA is absent in many gram-positive bacteria, implying alternative mechanisms that can facilitate the initial steps of pre-protein translocation through the Sec pathway. The chaperones prevent pre-proteins from aggregating and folding prematurely, ensuring compatibility for membrane transport. Furthermore, these chaperones interact with SecA to facilitate pre-protein transport through the Sec system (64).

SecA is an ATPase and the molecular motor that drives the translocation of pre-proteins through the SecYEG translocon (66). In *B. subtilis*, SecA is encoded by gene *div*, which was initially identified as a temperature-sensitive mutant defective in septum formation and later found to be the homolog of *E. coli secA* (23, 24). Structural, biochemical, and biophysical studies from several bacteria reveal that SecA is a highly dynamic multidomain protein that binds to multiple partners such as pre-proteins, SecYEG translocon, membrane phospholipids, ATP, and ribosomes (67, 68). SecA purified from cells forms a dimer (69), yet the functional significance of dimerization is not entirely clear (70–72). The N-terminal part of SecA consists of two nucleotide-binding domains, NBD1 and NBD2 (also known as IRA2), and a pre-protein binding domain (PBD, also known as pre-protein cross-linking domain). NBD1 and NBD2 compromise the DEAD motor domain, which forms the interface for ATP binding and hydrolysis (73, 74). The C-terminal part of SecA contains a C domain that can be further divided into four subdomains: the helical scaffold domain, the helical wing domain, IRA1, and the C-terminal linker domain (75). SecA recognizes pre-proteins by interacting with their signal peptides and certain sequences of the mature proteins (76–79). The signal peptide forms an α-helical structure that binds to a groove formed by PBD and IRA1 of SecA (79). The groove is relatively long, mostly hydrophobic, and surrounded by polar and charged residues. The hydrophobic character of the groove is highly conserved, while the amino acid residues that make up the groove are not (79). These features explain how SecA can promiscuously bind to a variety of signal peptides. SecA powers pre-protein translocation by repeated cycles of ATP hydrolysis and nucleotide exchange. The ATPase activity of SecA is strongly stimulated upon binding to SecYEG translocon and acidic phospholipids (80, 81). However, despite extensive research, the exact mechanisms of how SecA directs pre-protein translocation are not yet fully understood. Various models have been proposed, including power stroke model, Brownian ratchet model, push-and-slide model, and reciprocating piston model (66, 82). The detailed mechanisms remain to be addressed.

#### SecYEG, the protein-conducting pore

SecYEG complex forms a membrane channel, which is the central component of the protein translocation machinery. The complex is composed of three subunits—SecY, SecE, and SecG—that form the pore through which unfolded proteins translocate (83, 84). Structurally, in *B. subtilis*, SecY consists of 10 transmembrane domains and forms a clamshell-like channel, whereas SecE has one transmembrane domain connected to the cytoplasmic domain via an amphipathic helix. These domains of SecE are the major sites for interaction with SecY and provide stability and flexibility to SecY (85). The third component of the pore complex is SecG. Although SecG is not essential for growth and cell viability, a *secG* mutant of *S. aureus* significantly affects protein secretion (86). In addition, the deletion of *secG* results in the cold-sensitive phenotype of *B. subtilis* and also affects the extracellular protein secretion at lower temperatures, suggesting its importance as a part of the active SecYEG channel (87). Structurally, SecA and SecY are highly conserved across bacterial species, whereas SecE and SecG are less well conserved and shorter in *B. subtilis* and *S. aureus*, compared to the gram-negative counterparts (88). The SecYEG complex undergoes three conformational states—closed, partially open, and fully open—upon binding to the SecA-ATP complex. The signal sequences or transmembrane helices intercalate with the lateral gate of SecY to facilitate protein movement through the membrane (71, 89, 90). This highly dynamic equilibrium of the SecYEG complex assembly likely allows it to be adaptive to a wide range of substrates.

#### Auxiliary Sec components

The auxiliary components (SecDF-YajC) further enhance the efficiency of translocation in an ATP-independent way (91). Unlike *E. coli*, SecD and SecF in *B. subtilis* are present in a single open reading frame encoding for a single polypeptide, SecDF (92). It has 12 membrane insertion domains with N- and C-terminal cytoplasmic domains. Further studies revealed that SecDF is not essential for the growth and survival of *B. subtilis* under normal physiological conditions. However, mutations in *secDF* affect the secretion of protein AmyQ, indicating a role for the optimal transport of some proteins (93). The function of SecDF has been predicted to either stimulate the assembly of the Sec translocon or clear out the translocon from the misfolded pre-protein or signal peptides (94). In *E. coli*, it has been shown that SecDF forms a heterotrimeric membrane complex with a third component, YajC, that stimulates translocation by utilizing the proton motive force (95). A gene homologous to *E. coli yajC* has been identified in the genome of *B. subtilis*, designated as *yrbF*. Unlike *E. coli*, *secDF* and *yrbF* locate in the same chromosomal region but are not co-transcribed with *secDF* in *B. subtilis* (96).

#### SPase

Once the precursor protein engages with the SecYEG complex, the SPases (SPases I and II) located at the membrane cleave the signal peptides from the pre-proteins, releasing the mature proteins from the membrane (56). Bacterial type I SPase cleaves Sec-dependent signal peptides, whereas type II SPase is specific for processing lipoprotein precursors. Type I SPases show sequence homogeneity across both gram-positive and gram-negative bacteria. They have a short N-terminal cytoplasmic sequence followed by a transmembrane helix that aids their integration into the membrane, along with a C-terminal extracellular enzymatic domain (97). Despite the sequence similarity, structurally, the SPases in gram-positive bacteria differ from their gram-negative counterparts. For example, SPases in gram-positive bacteria are anchored to the membrane by one N-terminal transmembrane domain, whereas gram-negative SPases—including LepB, an SPase-I in *E. coli*—contain two N-terminal transmembrane segments (86, 98). Interestingly, multiple type I SPases have been observed within a single species of gram-positive bacteria. In *B. subtilis*, five paralogs of SPase-I have been found: SipS, SipT, SipU, SipV, and SipW, which are regulated under different regulons (99). SipS/T/U/V have overlapping substrate specificity, whereas SipW is more specific toward TasA, a crucial protein for biofilm matrix formation (100). *Staphylococcus* encodes two type I SPase homologs, SpsA and SpsB (55, 101). While SpsA is dispensable as it lacks two conserved catalytic residues, SpsB is an essential protein that operates using a Ser/Lys catalytic dyad mechanism, where Ser36 acts as the nucleophile and Lys83 as the general base (101,,^102^ ^102^,^102^). It cleaves the signal peptides from pre-proteins, including numerous virulence factors and growth-associated proteins. Interestingly, *S. aureus* can compensate for SpsB deficiency by upregulating a gene cassette encoding an ABC transporter. (103). Additionally, *spsB* depletion in *S. aureus* results in reduced processing of lipoteichoic acid synthase (LtaS) and loss of septal trafficking of cell wall anchored surface protein with a YSIRK signal peptide (see section “YSIRK/G-S Signal Peptide-Mediated Septal Trafficking”), correlating its crucial role in LTA biosynthesis and septal protein secretion (55, 104).

#### Signal peptide peptidase

In addition to the SPases, bacteria possess intramembrane site 2 proteases (S2Ps), known as signal peptide hydrolase or signal peptide peptidase (SPPase), that play crucial roles in quality control and folding stress response (105). Once the signal peptide is cleaved from the pre-protein by SPase, the cleaved remnants undergo further cleavage by S2Ps to release them from the membrane or to generate cleaved products for signal transduction (106). In *E. coli*, signal peptide peptidase A (SppA) removes the peptide fragments after SPase-mediated proteolysis (105, 107). Similarly, in *B. subtilis*, two SppA-like proteins have been characterized, SppA and TepA (108). While SppA is a membrane protease involved in protein secretion, TepA appears to be a cytoplasmic protein and is required for early-stage efficient precursor protein processing. Additionally, *B. subtilis* expresses a membrane-bound signal peptide hydrolase RasP, a homolog of *E. coli* RseP, that binds to the signal peptides processed by SPase I and further cleaves them (109, 110). While RasP is an intramembrane protease that cleaves the peptides in the membrane plane, SppA can only cleave the signal peptides that are either released or extracted from the membrane (105, 111). Homolog of RasP/Eep in *S. aureus* has been shown to affect the production of sex pheromone cAM373_SA and the secretion of a number of proteins (112, 113). Interestingly, *rasP* deletion in *S. aureus* triggered increased production of SPase SpsB (112, 113). Proteomics study of *S. aureu*s culture supernatant revealed the release of C-terminal signal peptide fragments of 18 secreted proteins, suggesting additional cleavage of signal peptides beyond SpsB may occur; however, their biological significance and the involvement of SPPase are unclear (114).

## PROTEIN PROCESSING IN THE MEMBRANE-WALL INTERFACE: A JOURNEY FOR SURVIVAL

Most newly synthesized proteins are transported across the cytoplasmic membrane in an unfolded state. Once they emerge from the Sec translocase into the extracellular space, they encounter several factors and chaperones, which help the proteins fold and mature into their active forms before they are secreted. Unlike gram-negative bacteria, which have a well-defined periplasmic space, gram-positive bacteria have a membrane-wall interface (see section “Introduction”) with a high density of immobilized negative charge (115). The processing of the proteins in the membrane-wall interface is crucial for maintaining their activity and functionality in the challenging extracellular environment (116).

### Chaperones, the guiding hands

Post-translocational folding of proteins requires extracellular chaperones. One of the most prominent chaperones is PrsA (parvulin-type peptidyl-prolyl *cis*/*trans* isomerase), a membrane-bound foldase present ubiquitously in gram-positive bacteria (117). PrsA catalyzes *cis*-*trans* isomerization of peptide bonds preceding proline residues and is thought to assist folding of proteins in the membrane-wall interface (118). The *prsA* gene in *B. subtilis* was initially identified from screening for mutants deficient in AmyQ α-amylase secretion (119). PrsA is essential for the survival of *B. subtilis* under normal growth conditions, yet a null *prsA* mutant is viable in the presence of a high concentration of magnesium (120, 121). PrsA is a membrane-bound lipoprotein. Sequence analysis reveals that *B. subtilis* PrsA features an N-terminal domain, a central parvullin-like PPIase domain, and a C-terminal domain (122). All three domains are necessary for PrsA function in protein secretion and viability. However, amino acid mutations of the conserved active site in the PPIase domain abolish PPIase activity but do not significantly influence protein secretion or bacterial growth (122). Structural analysis of *B. subtilis* PrsA demonstrates that its central PPIase domain is inserted into a composite N- and C-terminal chaperone-like domain (123). Despite lacking sequence homology, the NC chaperone-like domain is structurally similar to SurA, a periplasmic chaperone in *E. coli* (123). Moreover, PrsA forms a dimer of a large, bowl-like shape, which is thought to be required for *in vivo* chaperone activity (123). Combined with the data from mutation and deletion studies in *B. subtilis* and other gram-positive bacteria, it is proposed that the NC domain carries essential chaperone functions, whereas the PPIase activity of PrsA is relevant for a subset of substrates (123). In *S. aureus*, a *prsA* mutant is viable. A *prsA* mutant in a community-acquired methicillin-resistant *S. aureus* (CA-MRSA) strain USA300 exhibited reduced protease, phospholipase, and alpha-toxin activity but only affected the secretion of a small number of proteins and did not affect virulence in a murine abscess or sepsis model (124). In contrast, a *prsA* mutant in a methicillin-sensitive lab strain, HG001, was shown to affect the secretion of a large number of secreted and cell wall-associated proteins and significantly attenuated in a murine sepsis model (125). The apparent discrepancy could be explained by the different strain background used in these studies. PrsA also assists in the folding of membrane proteins, such as penicillin-binding proteins, which in turn contributes to antibiotic resistance (120, 126). Deletion of *prsA* in *S. aureus* decreases the amount of PBP2a in the membrane and makes methicillin-resistant *S. aureus* more sensitive to the β-lactam antibiotics (126, 127). In other gram-positive species, such as *Bacillus anthracis*, more than one PrsA homolog has been identified through genetic screening (PrsA1 or PrsAA, PrsA2 or PrsAB, and PrsA3 or PrsAC) (128). Another gram-positive pathogen, *Listeria monocytogenes*, possesses two homologs of PrsA: PrsA1 and PrsA2 (129–131). Expression of *prsA1* does not complement a *prsA2* deletion, suggesting that they have non-redundant functions (132). Sequence analysis of PrsA homologs from gram-positive bacteria reveals that the PPIase domain is highly conserved, while the NC chaperone domain varies (123). It is proposed that the conservation of the PPIase domain is necessary for the enzymatic function, and the variation in the NC domain may correlate with different substrate specificities (123).

### Quality control by HtrA family proteases and chaperones

Misfolded proteins post-translocation induce stress in bacteria, prompting either proper folding or degradation of anomalous ones. Extra-cytoplasmic proteases are well studied in *E. coli,* where DegP, a member of HtrA (High temperature requirement) family of serine proteases, functions as a molecular chaperone at low temperature and protease at high temperature in the periplasm (133). *E. coli* DegP contains an N-terminal signal peptide for its periplasmic localization, a catalytic protease domain, and two PDZ domains (134). PDZ1 of DegP binds to a C-terminal motif of the substrate, and the active site of DegP binds to substrate sequence around the cleave site (135–137). Substrate binding triggers conformational changes and leads to the assembly of proteolytically active large polyhedral DegP cages (12- or 24-mers) (135, 138). Compared to the gram-negative counterparts, the DegP/HtrA homologs in many gram-positive bacteria are membrane-anchored by having a transmembrane domain instead of a signal peptide; moreover, the gram-positive HtrA homologs contain only one PDZ domain (139–142). Sequence analysis identified three homologs of HtrA—HtrA (YkdA), HtrB (YvtA), and YycK—in *B. subtilis*, but only HtrA and HtrB are responsive to heat and secretory stress, whereas YycK is insensitive to these stimuli (143, 144). Furthermore, the expression of *htrA* and *htrB* in *B. subtilis* is upregulated by the two-component system, CssRS, which detects the extracytoplasmic secretion stress, such as overproduction of secreted alpha-amylases or elevated temperature (145–148). Proteomics studies demonstrate that *B. subtilis* HtrA and HtrB not only respond to secretion stress but also perform protease and chaperone activities under non-stress conditions, crucial for the secretion and activities of many exoproteins in *B. subtilis* (149). *S. aureus* encodes two HtrA-like proteins, namely, HtrA1 and HtrA2. Their functions have been studied in two *S*. *aureus* strains: RN6390, a methicillin-sensitive strain defective in alternative stress response factor σ^B^ signaling, and COL, a methicillin-resistant strain with a functional σ^B^ (150). The *htrA1* and *htrA2* single and double mutants are thermosensitive in strain COL but not in RN6390. The double mutant of *htrA1*&*htrA2* drastically altered the exoprotein profile of RN6360, reduced virulence factors in the culture supernatant, and diminished virulence in a rat model of endocarditis (150). However, single mutants of *htrA1* and *htrA2* did not show strong phenotypes in strain COL (150). The results suggest that HtrA proteins play different roles in different strains, which could be impacted by σ^B^ regulation. The exact functions and regulation of HtrA proteins in *S. aureus* remain unclear.

### Post-translocational modification by thiol-disulfide oxidoreductases

Unlike gram-negative bacteria, where thiol-disulfide oxidoreductases contribute to the post-translational modification of the transported proteins in the periplasm, little is known about disulfide modification of the transported proteins in gram-positive bacteria. Four disulfide-oxydoreductases, BdbA, BdbB, BdbC, and BdbD (Bdb stands for Bacillus disulfide bond formation) have been identified in *B. subtilis* and they work in pairs, i.e., BdbA/B and BdbC/D, similar to *E. coli* DsbA/DsbB (151, 152). While the small, soluble BdbA lacks a membrane-spanning domain and is involved in modification of specific proteins such as lantibiotic sublancin, BdbB and BdbC possess four predicted transmembrane domains and are crucial for folding and stability of alkaline phosphatase. A detailed crystal structure of BdbD revealed that its TDOR domain (thiol-disulfide oxidoreductase) is linked to the membrane via the N-terminal transmembrane segment and interacts with the substrate like apo-cytochrome C (151). In addition to these enzymes, *B. subtilis* possesses two additional cytoplasmic TDORs (ResA and StoA) required for cytochrome C maturation and endospore biogenesis, respectively, and they reduce the disulfide bonds in their substrates created by BdbD (152–154). Alongside protein factors, metal ions like Ca^2+^, Fe^3+^, and Mg^2+^ influence the folding and stability of some secreted proteins in the extracellular space by directly binding to the proteins or affecting the overall net charge of the cell wall (116, 155–157).

## PROTEIN TRANSPORT ACROSS THE CELL WALL: NAVIGATING TOWARD THE DESTINATION

Transport across the thick peptidoglycan layer in gram-positive organisms involves both passive and facilitated mechanisms (158). Once secreted, proteins may remain attached to the cell surface either covalently or non-covalently. These mechanisms enable the dynamic distribution of cell-surface-associated proteins during cell wall remodeling and growth (159).

### Covalent binding to the cell wall: the act of sortase

Sortases are transpeptidase enzymes that anchor surface proteins to the bacterial cell wall peptidoglycan. The activity of sortase enzymes was first studied in the early 1990s by Schneewind and colleagues, and the first isolated sortase was sortase A (SrtA) from *S. aureus* (160, 161). The substrates of sortases are synthesized in the cytoplasm with an N-terminal signal peptide and a C-terminal cell wall sorting signal (162). The cell wall sorting signal contains an LPXTG motif, a hydrophobic domain, and a positively charged C-terminal tail (163). The N-terminal signal peptide is recognized by SecA and subjected to SecYEG translocon (164). Upon membrane translocation, the signal peptide is cleaved by type I SPase, and the rest of the protein remains transiently attached to the membrane via a C-terminal cell wall sorting signal. SrtA recognizes and cleaves the LPXTG motif between threonine (T) and glycine (G) and covalently links surface protein precursors to the pentaglycine cross-bridge of lipid II, which is ultimately incorporated into mature peptidoglycan through transglycosylation and transpeptidation reactions (165). Many gram-positive bacteria encode sortase enzymes, which fall into six groups (sortase A–F) based on phylogenetic analysis (166). In *B. subtilis*, two putative class D sortases, YhcS and YwpE, have been identified along with two surface proteins: a nuclease YhcR with an LPDTS motif and a nucleotidase YfkN harboring LPDTA motifs (167–169). While YhcR has been confirmed as a substrate of YhcS, little is explored about YwpE. Furthermore, human pathogen *B. anthracis* encodes three sortase proteins, SrtA/B and SrtC of class A, B, and D. *B. anthracis-*SrtA is responsible for anchoring multiple *B. anthracis* phage receptor proteins, including GamR, BasB, BasE, and BasJ (170). In *S. aureus*, class A sortase, SrtA is the housekeeping sortase that anchors cell surface proteins to the cell wall, which are important for bacteria-host interactions during pathogenesis (171). Additionally, staphylococcal SrtB, a class B sortase, recognizes NPQTN cell sorting motif and is specialized in aiding bacteria adaptation to iron-limited environment by anchoring the iron responsive surface determinant (172). SrtB is widely present in *Bacillus*, *Listeria*, and *Staphylococcus*. Class D sortase, SrtC, is present in both low- and high-GC gram-positive bacteria. In *B. anthracis*, SrtC recognizes LPNTA at the C-terminus of the substrates BasH and BasI that localize in the forespore and divisional plane, respectively (173). Beyond these groups, sortase D and sortase F mainly facilitate the pillin assembly on the cell surface (174, 175). Class E sortase, SrtE, is found in *Corynebacterium* sp., *Streptococcus* sp., and *Actinomyces* sp. and recognizes LAXTG or LPXTG motif (170). The sortase-mediated protein anchoring to cell walls plays key roles in bacteria-host interactions, including host cell invasion, adhesion, and receptor signaling, ultimately shaping bacterial pathogenesis (162).

### Travel across the cell wall: more than a passive journey

The bacterial cell wall is a porous structure that allows secreted proteins to diffuse. In 1996, a study by Demchick and Koch demonstrated that *B. subtilis* peptidoglycan is permeable to globular proteins of size 25 kDa (176). Interestingly, many proteins secreted by *B. subtilis* exceed 25 kDa in size and are not globular, indicating that transport across the bacterial cell wall is not an uncontrolled passive process (158). Further studies revealed that the extracellular release of certain enzymes, including α-amylase, levansucrase, and subtilisin from *Bacillus* sp., requires additional controlling factors such as the growth phase, rate of protein folding, interaction with cations, the net charge of the cell wall, interaction with chaperones, and pH of the extracellular environment (158). A recent study by Strach et al. provides evidence that the AmyE secretion in *B. subtilis* occurs at defined regions within the cell wall termed as “secretion zones,” which act as diffusion pathways allowing AmyE secretion across the cell wall (177). Another recent work suggests that entropy drives directional transport of large proteins across the cell wall (178). An actin nucleation-promoting and virulence factor, ActA, in *L. monocytogenes* has been found to remain attached to the membrane, while its N-terminus is exposed through the cell wall (179). A biochemical study and biophysical model revealed that proteins with at least 500 amino acids of disordered regions require entropy-driven translocation, which depends on the cell surface geometry and protein length (178). Most of the exogenous disordered proteins in gram-positive bacteria, including *S. aureus*, *L. monocytogenes*, and *B. subtilis*, do not require any specific sequences or polypeptide orientations to translocate across the cell wall. Currently, our knowledge of these processes is limited.

### Non-covalent attachment: a symphony of binding motifs

Some cell surface-displayed proteins are non-covalently attached to the cell surface via specific binding motifs such as choline-binding motif, LysM motif, GW domain, S-layer homology motif, CWB2, and SH3b domain. Choline-binding motif is also known as cell wall binding domain type I, which includes several imperfect repeats of 20 amino acids with some conserved aromatic amino acids. *Streptococcus pneumoniae* autolysin, LytA, and virulence-related pneumococcal surface proteins PspA and PspC share a C-terminally located choline-binding motif, which interacts with the choline group of teichoic acid (180). CwlB, a *B. subtilis* autolysin, and *C. difficile* adhesion protein Cwp66 have an N-terminal cell wall binding domain type II, CWB2, which is 100 amino acids long and binds to the S-layer on the cell wall (181, 182). Another specific cell wall binding motif is the LysM domain, which is 40 amino acids long present in Archaea, gram-negative, and low and high GC gram-positive bacteria. This domain has been reported to bind to the N-acetylglucosamine of peptidoglycan (32, 183). *S. aureus* secretes murein hydrolases, including LytN and Sle1, with LysM domain. Mutational analysis revealed that the LysM domain is crucial to localize these proteins to the septal peptidoglycan (cross-wall) for daughter cell separation (184). Another cell wall binding motif is the GW motif, which is ~80 amino acid long with a Gly-Trp repeat. GW motifs are mostly seen in autolysins (185). An invasion protein, InlB, in *L. monocytogenes* uses its GW motif to interact with the lipoteichoic acid to remain attached to the cell surface during host-pathogen interaction (186). In addition, S-layer homology domains (SLHDs) consisting of ~50 amino acids are frequently present in either N- or C-terminal end of the surface displayed proteins. SLHD does not bind to the peptidoglycan directly but non-covalently binds to the secondary cell wall polymers, which allows a dynamic attachment-detachment mode. Proteins bearing this motif are mostly amidases, peptidases, and proteases (185). SH3b domains are found in staphylococcal phage endolysins and some autolysins. This domain recognizes and binds to the glycine cross-bridges in the peptidoglycan (187). Binding to the cell wall via specific repeat sequence is common in bacterial autolysins. For instance, Atl is the most predominant autolysin found in *Staphylococcus,* which has multiple repeats between its catalytic domains (188). The repeats of autolysin (R1, R2, and R3) are responsible for targeting Atl to the cross-wall for daughter cell separation. Wall teichoic acid has been shown to restrain Atl binding to the cross-wall, whereas lipoteichoic acid binds to the repeats of Atl (189–192). Besides motif-mediated binding, proteins can attach to the bacterial cell surface through charge or hydrophobic interaction. Streptococcal enzymes like GAPDH and α-enolase are attached to the cell wall via hydrophobic interaction, which can be disrupted by the addition of chaotropic agents (193). Also, a virulence factor, PspA, in *S. pneumoniae* forms charge interaction between its C-terminal repeat region and the cell wall (194). In summary, secreted proteins may remain attached to the cell surface via specific or non-specific interactions.

## DYNAMIC SUBCELLULAR LOCALIZATION OF PROTEIN TRANSLOCATION SYSTEMS

Cumulative data from the literature suggest that protein translocation across the cell envelope occurs at specific sites and often correlates with cell division and cell growth phase. The dynamic and specific localization not only supports efficient substrate delivery and their functionality but also provides mechanisms that coordinate protein secretion with other cellular functions such as metabolism, cell division, and cell wall synthesis (195). In this section, we will focus on the dynamic localization of protein translocation systems and their substrates.

### Protein secretion and processing machinery

The localizations of the Sec machinery vary among species. SecA was initially reported to concentrate at a single microdomain termed “ExPortal” in group A *Streptococcus S. pyogenes* (196). Yet conflicting data from another study showed that SecA is distributed throughout the membrane (197). The microdomain localization of SecA was also found in *Enterococcus faecalis* and *Streptococcus mutans* (198, 199). In *S. pneumoniae*, SecA and SecY exhibit cell cycle-dependent localization: SecA localizes at the septum during early cell division and distributes to peripheral sites during late cell division (200). In group B *Streptococcus S. agalactiae*, SecA predominantly localizes at the septum (201), while in *B. subtilis*, it follows a spiral-like pattern along the cytoplasmic membrane, aligning with the cytoskeletal protein MreB/Mbl and the site of cell wall synthesis (202). *S. aureus* SecA was found to be distributed throughout the plasma membrane but not restricted to the septum (164). A recent study using single molecular tracking microscopy revealed detailed movement of *B. subtilis* SecA with three types of mobility states: a free diffusive independent movement in the cytoplasm, a medium movement likely ribosome bound, and a static mode probably engaged with active SecYEG translocons located at the membrane (58). In addition to Sec machinery localization, a recent study revealed that *S. aureus* SpsB is enriched at the septum, and its depletion results in an abnormal cell separation phenotype, suggesting a dual role of SpsB in protein secretion and cell cycle regulation (55). All these findings indicate dynamic localizations of the Sec system across species, which often correlates with cell division and areas of high metabolic or cell wall biosynthesis activity (199–201, 203).

Protein processing in the periplasmic space and anchoring to the cell wall often co-localize with the Sec machinery and associate with the cell cycle. In *S. pyogenes*, HtrA has been shown to colocalize with SecA in the ExPortal near the division septum (204). *S. pneumoniae* HtrA localizes at the cell equators and division septum, largely coinciding with SecA-SecY localization (200). The localization of SrtA has been studied in several gram-positive bacteria. In *S. pyogenes*, SrtA is recruited to the septum at the early stage of cell division and localizes as distinct foci mostly at the septum (205). In *E. faecalis* and *S. mutans*, SrtA co-localizes with SecA as single foci mostly found at the septum and the polar region, while in *S. agalactiae*, SrtA co-localizes with SecA at the septum (198), (199, 201). In contrast, SrtA is distributed over the entire cell surface in *S. pneumoniae*, suggesting more dispersed protein anchoring (200). Though SrtA localization in *S. aureus* has not been reported, the localization of surface proteins at both septum and cell periphery suggests a cell cycle-dependent localization of SrtA (32, 206). YhcS in *B. subtilis* localizes as a helical pattern resembling the pattern observed for the cytoskeletal protein MreB/Mbl, indicating a possible interaction between sortase and the cell wall synthesis machinery (**207**). Despite extensive studies, the mechanisms governing the subcellular distribution of the Sec system, HtrA, and sortases remain largely unknown.

### YSIRK/G-S signal peptide-mediated septal trafficking

As mentioned above, the amino acid sequences of signal peptides are highly variable. A conserved sequence in a signal peptide often suggests specialized secretion. Initially discovered in staphylococcal lipases, a special group of signal peptides, termed YSIRK/G-S signal peptide, was found in many cell wall-anchored proteins and some secreted proteins (208, 209). The YSIRK signal peptides contain a highly conserved YSIRK/G-S motif (YSIRKxxxGxxS) positioned at the beginning of the hydrophobic core of the signal peptide. The YSIRK signal peptides are widely distributed in gram-positive bacteria and most abundant in *Staphylococcus*, *Streptococcus*, and *Lactobacillus* (InterPro: IPR005877, Pfam: PF04650). Many of the YSIRK proteins are known to be important virulence factors (205, 210). Earlier work demonstrated that the YSIRK signal peptide directs septal secretion and subsequent cross-wall anchoring (197, 205, 210, 211). Further studies have demonstrated that *S. aureus ltaS* mutant deficient in LTA synthesis no longer restricts the secretion of YSIRK proteins into septal membrane (164). Subsequently, LTA is shown to accumulate at the staphylococcal cell periphery but is barely detectable at the septum (32). The extracellular enzymatic domain of LTA synthase, LtaS, is cleaved by signal peptidase SpsB (104). A recent study shows that SpsB is enriched at the septum, which spatially restricts LtaS-mediated LTA synthesis (55). However, several mechanisms appear to influence the interactions among SpsB, LtaS processing, and YSIRK protein trafficking. First, MspA, a factor that interacts with both LtaS and SpsB, may limit LtaS processing (212); second, LtaS processing by SpsB seems to be triggered upon binding of LtaS to the LTA lipid anchor (213); third, and possibly not last, SpsB is enriched at the septum, where it also processes YSIRK proteins (55). Cells depleted of *spsB* and *mspA* exhibit cell cycle arrest and pronounced defects in cell separation, phenotypes that are likely to be contributed by the dysregulated septal LTA assembly (55, 212). Collectively, these findings indicate intimate connections between protein trafficking and cell cycle, cell division, and cell envelope biogenesis, which provide new directions for future investigation.

## CLOSING REMARKS

Since the initial discoveries in 1990, extensive research has significantly deepened our understanding of protein transport in gram-positive bacteria. However, there are still many unknowns as mentioned above. An extensive understanding of the protein trafficking process offers great opportunities for both therapeutic and industrial applications. Indeed, tremendous effort has already been made. Many of the enzymes discussed above have been used as drug targets (141, 214, 215). Genetic engineering of secretion systems is promising to yield large-scale synthesis and secretion of industrially valuable bioproducts (56). Owing to significant advancements in the toolbox to study protein translocation (216), such as biochemical approaches, genome sequencing and bioinformatics, proteomics, protein structural analysis, super-resolution microscopy, and singlemolecule tracking, we are excited to see continued discoveries in this research area.
